# Behçet's Syndrome With Multiple Bilateral Pulmonary Aneurysms Associated With Endomyocardial Fibrosis Presented With Pulmonary Emboli: A Case Report

**DOI:** 10.7759/cureus.66281

**Published:** 2024-08-06

**Authors:** Ali H Almutamaiz, Sultan A Alshoabi, Eman S Al Akhali, Abdullgabbar M Hamid, Awadia Gareeballah, Awatif M Omer

**Affiliations:** 1 Department of Radiology, Al-Thawra Modern General Hospital, Sanaa, YEM; 2 Department of Radiology, Faculty of Medicine, Sana'a University, Sanaa, YEM; 3 Department of Diagnostic Radiology, College of Applied Medical Sciences, Taibah University, Al-Madinah Al-Munawwarah, SAU; 4 Department of Radiology, Advanced Al Razi Diagnostic Center, Sanaa, YEM; 5 Department of Radiology, Rush University Medical Center, Chicago, USA

**Keywords:** endomyocardial fibrosis, budd-chiari syndrome, pulmonary artery thrombosis, multiple pulmonary artery aneurysms, hughes-stovin syndrome, behçet's syndrome

## Abstract

Behçet's syndrome (BS) is a rare chronic multisystemic inflammatory disorder of unknown etiopathogenesis. BS is classified as a vasculitis of variable vessel size, which can manifest in both arterial and venous blood vessels. BS commonly presents with mucocutaneous and ocular manifestations. Superficial and deep vein thrombosis is present in 50% of patients, with atypical venous thrombosis affecting the inferior vena cava, superior vena cava, hepatic veins with Budd-Chiari syndrome, portal vein, cerebral sinuses, and right atrium and ventricle. Arterial manifestations include in situ thrombosis, pulmonary artery aneurysms, aneurysms of the abdominal aorta, and aneurysms of visceral and peripheral arteries. This article reports a new case of BS in a 28-year-old female patient who presented with severe dyspnea and hemoptysis. Echocardiography and cardiovascular magnetic resonance imaging led to the diagnosis of endomyocardial fibrosis and a large right ventricular thrombus with pulmonary embolism. Computed tomography angiography revealed multiple pulmonary aneurysms and emboli. Rare findings such as endomyocardial fibrosis and Budd-Chiari syndrome were noted. This case highlights the role of medical imaging modalities in diagnosing rare syndromes such as BS, as demonstrated in the current case.

## Introduction

Behçet's syndrome (BS), also known as Silk Route disease, is a rare chronic multisystemic inflammatory disorder characterized by a relapsing and remitting course [1,2]. BS is prevalent along the Silk Road, with incidence rates of 20-420/100,000 in Turkey, 80/100,000 in Iran, and 0.64/100,000 in the United Kingdom [3]. BS has an unknown etiopathogenesis and commonly presents with mucocutaneous and ocular manifestations. Necrotizing vasculitis in BS involves variable vessel size, which can manifest in both arterial and venous blood vessels [4]. Superficial and deep vein thrombosis occurs in 50% of patients, with atypical venous thrombosis affecting the inferior vena cava (IVC), superior vena cava (SVC), hepatic veins with Budd-Chiari syndrome (BCS), portal vein, cerebral sinuses, and right atrium and ventricle. Arterial manifestations include vasculitis, which manifests as in situ thrombosis, pulmonary artery aneurysms, and aneurysms of the abdominal aorta, as well as visceral and peripheral arteries [5].

The diagnosis of BS was first described by Turkish dermatologist Hulusi Behçet as a recurrent triad of oral ulcers, genital ulcers, and uveitis. International consensus recommendations for the diagnosis and management of BS have since been established [6]. The frequency of organ involvement in BS is cardiovascular (45.3%), gastrointestinal (42.3%), neurologic (21.8%), and thoracic (8.5%) [7].

This case report aims to document a new case of BS presenting with an endocardial thrombus, recurrent pulmonary emboli, and multiple pulmonary artery aneurysms. It also presents very rare findings such as endomyocardial fibrosis and BCS. The case highlights the role of medical imaging modalities in guiding the diagnosis of rare syndromes such as BS.

## Case presentation

A 28-year-old Middle Eastern female patient with no previous chronic health problems complained of deep venous thrombosis (DVT) one week after a normal vaginal delivery. She received the proper treatment for DVT.

After 10 months, she started complaining of severe dyspnea with hemoptysis and visited the emergency room (ER). The patient was fully conscious and oriented. Her blood pressure was 100/80 mmHg, with a heart rate of 80 beats per minute, a respiratory rate of 15 cycles per minute, and a temperature of 37.2°C.

**Figure 1:**
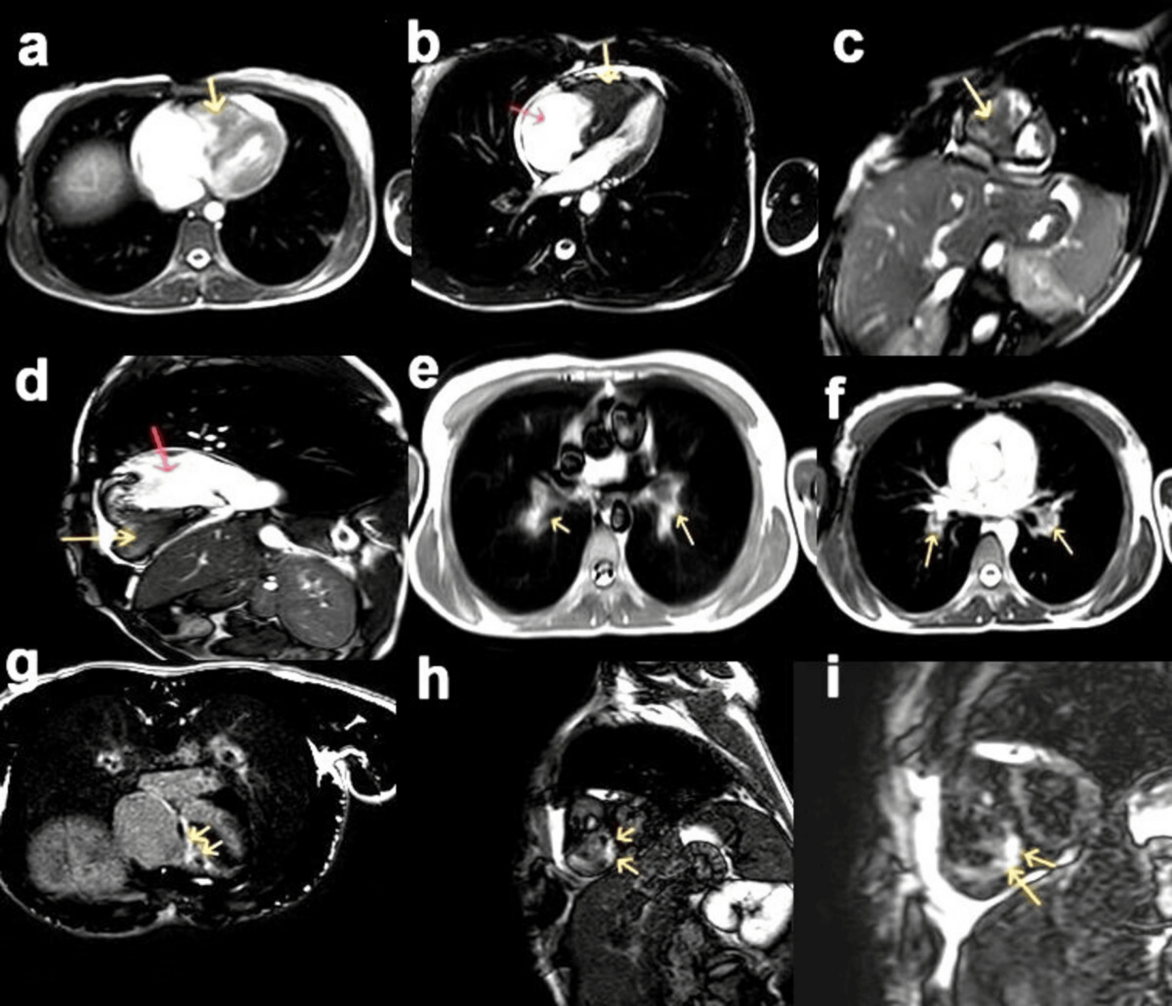
Cardiac magnetic resonance (CMR): (a, b) Localized and cine four-chamber views and (c, d) short-axis and two-chamber views of the right side show a dilated right atrium (RA) (red arrow), small right ventricle (RV) cavity with a thrombus filling the small lumen (yellow arrow), and a small pericardial effusion. (e, f) Axial views noted bilateral pulmonary artery dilatation with a filling defect. (g, h, i) Late gadolinium enhancement images reveal V-shaped subendocardial enhancement of the RV wall extending into the inflow tract (arrows).

In the ER, an echocardiogram (Echo) was performed, revealing a reduced right ventricular (RV) cavity volume filled with a suspicious mass-like structure, and a dilated right atrium (RA). The patient was scheduled for cardiac magnetic resonance (CMR) imaging, which demonstrated the dilated RA and apical obliteration of the right ventricle. The RV mass-like structure did not enhance, consistent with the thrombus. A small right ventricle cavity with V-shaped delayed phase subendocardial enhancement at the apex was also noted, consistent with the diagnosis of endomyocardial fibrosis with a large RV thrombus. Incidental bilateral pulmonary emboli were also noted (Figure 1).

**Figure 2:**
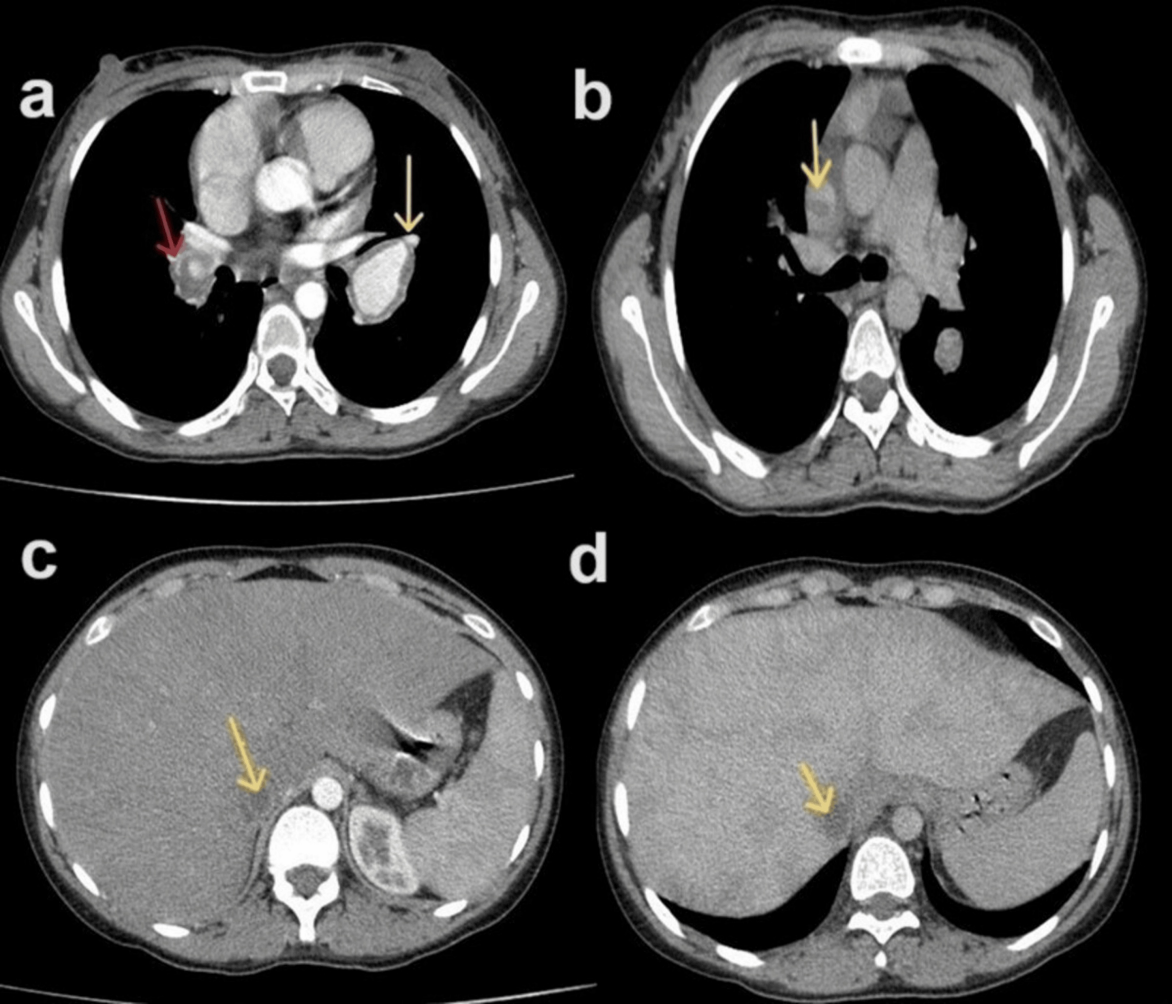
Computed tomography pulmonary angiography (CTPA): (a) Arterial phase shows right pulmonary artery aneurysmal dilatation with a filling defect representing thrombosis (red arrow) and left pulmonary artery dilatation with wall thickening (yellow arrow). (b) The late venous phase shows a filling defect in the superior vena cava (SVC), indicating SVC thrombosis (yellow arrow). (c) Arterial phase and (d) late venous phase show inferior vena cava (IVC) filling defects (yellow arrows) with hepatomegaly and heterogeneous enhancement (nutmeg liver), indicating an association with Budd-Chiari syndrome.

The patient was scheduled for computed tomography pulmonary angiography, which demonstrated bilateral pulmonary artery aneurysmal dilation and thrombosis. Thrombosis in the SVC and IVC were also detected by CT (Figure 2).

In the intensive care unit (ICU), blood investigation revealed elevated C-reactive protein and low hemoglobin. The white blood cells and the differential count were at normal levels. Platelet count and other coagulation profile tests were normal. No neurological deficits or ocular lesions were noted. There were no signs of infection, which excluded the possibility of an infectious process such as fungal or bacterial infection as a potential cause of the patient's multiple aneurysms and thrombosis. Additionally, there were no signs of a genetic disorder such as Ehlers-Danlos syndrome. No neurological symptoms were developed. Four months after the initial presentation, oral ulcers were developed. The patient was diagnosed with Behçet's disease based on the clinical and radiological findings.

No specific antibodies were detected. In the ICU, the patient suffered several attacks of acute on chronic pulmonary embolisms while under thrombolytic treatment and anti-autoimmune medication. Despite this treatment, the RV thrombus remained a source of emboli.

After five months in the ICU, Echo revealed persistent enlarged RA with changes suggesting severe pulmonary hypertension. The RA thrombus was not visualized. Lab investigation revealed an international normalized ratio (INR) of 2 (normal INR = 0.8-1.1), prothrombin time (PT) of 24.2 (normal PT = 11-13.5 seconds), and partial thromboplastin time (PTT) of 72.5 (normal PTT = 25-35 seconds). The patient was discharged with mild improvement with the following treatment: augmentin tablet 1 gm (1 x 2 x 7); warfarin 3 mg tablet (1 x 1), diltiazem tablet 60 gm (1 x 2), azathioprine tablet 50 gm (1 x 1), citalopram tablet 10 mg, and prednisolone tablet 20 mg (1 x 1). An outpatient follow-up was scheduled a month after discharge.

## Discussion

BS is a rare condition with various phenotypes of unknown etiology and a wide range of clinical manifestations secondary to chronic inflammation and vasculitis, which can often create a diagnostic dilemma. BS usually affects young males of Mediterranean, Middle Eastern, or Asian ethnicity. BS has a wide range of clinical manifestations, including oral aphthosis in over 95% of cases, genital aphthosis in 60-90%, pseudofolliculitis/erythema nodosum in 40-90%, uveitis/retinal vasculitis in 45-90%, gastrointestinal symptoms such as diarrhea, hemorrhage, perforation, and pain in 4-38%, venous/arterial thrombosis and aneurysms in up to 50%, neurological manifestations in up to 38.5%, and arthralgia/arthritis/ankylosing spondylitis in up to 93% [3]. In 2013, an international team (from 27 countries) proposed criteria that were felt to increase the sensitivity of Behçet’s disease diagnosis while maintaining reasonable specificity. In this proposal, the ocular, oral, and genital lesions are each given two points, while central nervous system involvement, skin lesions, and vascular manifestations are assigned one point each. The pathergy test, if used, is assigned one point. A patient scoring more than or equal to four points is classified as having Behçet’s disease [8].

In the present case report, the patient was a young Middle Eastern female who initially presented with DVT after delivery. This presentation is reported to be the most common form of single major vascular disease in BS, occurring in 67% of cases [9]. In the present case report, however, this presentation did not initially suggest a diagnosis of BS. This can be explained by the synchronization of DVT with the postpartum period, which diverted suspicion away from BS. Pulmonary artery thrombosis has also been reported as the first presentation of BS in another patient with BS [10]. Additionally, a previous BS case series reported pulmonary embolism, a rare and serious finding in BS, present in 1-10% of patients [11,12]. In the present case report, the patient earlier presented with DVT, which was treated. But now she presented with symptoms of pulmonary artery thrombosis in the form of severe dyspnea and hemoptysis. Echo and cardiac MRI confirmed a large right ventricular thrombus, an uncommon finding in BS [13], and multiple pulmonary thrombi/emboli.

Consistent with this case report, many cases are reported with the involvement of pulmonary artery thrombosis, IVC, and right hepatic vein thrombosis, representing an atypical vascular manifestation of BS [14]. In the same context, intracardiac thrombus, left anterior descending artery obstruction causing myocardial infarction, pulmonary artery aneurysm with in situ embolism, and suspected epiglottitis were reported as atypical life-threatening cardiopulmonary manifestations in pediatric BS cases [15]. In the current patient, multiple filling defects were seen in the SVC and IVC with hepatomegaly with heterogenous hepatic enhancement in nutmeg pattern suggesting BCS. These findings are compatible with a previous case report of BS associated with BCS, SVC, and IVC thrombosis [16]. A previous study reported that the coexistence of BS and BCS, and BS with BCS and IVC thrombosis, has a higher mortality rate than those without [17]. The mortality rate of BS is 3-4%, but the development of BCS with BS has a 61% mortality rate [18].

Hughes-Stovin syndrome, first described in 1959, involves cases presenting with deep venous thrombosis coupled with pulmonary artery mural thrombi and multiple aneurysms of the pulmonary arteries [19]. Initially, our team considered this diagnosis due to the absence of mucocutaneous manifestations. However, the associated cardiac findings on MRI, including a small right ventricle cavity with apical subendocardial enhancement extending to the inflow tract but sparing the outflow tract, led to the diagnosis of endomyocardial fibrosis. This has been reported in multiple previous cases to be associated with BS [20,21]. Cardiac lesions associated with BS include endocarditis, pericarditis, intracardiac thrombosis, myocardial infarction, endomyocardial fibrosis, and myocardial aneurysm [22]. Later, in the course of the patient's illness, oral ulcers developed. Additionally, many authors consider Hughes-Stovin syndrome to be a limited form or an early presentation of BS [23], which increased the treating team's confidence in the BS diagnosis.

In this patient, the presence of BCS with IVC and SVC thrombosis is a poor prognostic indicator, and she has spent five months in the ICU at the time of this report. The mortality rate of BS is 5%, with higher rates associated with male gender, arterial involvement, and the number of flares [24]. Future directions include genome-wide studies to understand the etiology of BS and subsequent trials to prevent and treat the disease.

## Conclusions

This case underscores the diagnostic challenges and severe complications of BS. The patient's progression from postpartum DVT to severe cardiopulmonary involvement highlights the importance of considering BS in atypical presentations and the crucial role of advanced imaging. The high morbidity and mortality associated with BS, particularly with conditions like bilateral pulmonary thrombosis, right ventricular thrombosis, and BCS, emphasize the need for prompt diagnosis and targeted treatment.
